# PM_2.5_ exposure increases dry eye disease risks through corneal epithelial inflammation and mitochondrial dysfunctions

**DOI:** 10.1007/s10565-023-09791-z

**Published:** 2023-02-14

**Authors:** Donghui Yu, Wenting Cai, Tianyi Shen, Yan Wu, Chengda Ren, Tingting Li, Chengyu Hu, Meijiang Zhu, Jing Yu

**Affiliations:** Department of Ophthalmology, Shanghai Tenth People’s Hospital, Tongji University School of Medicine, Shanghai, China

**Keywords:** Air pollutant, Particulate matters 2.5, Dry eye disease, Inflammation, Nrf2, NF-κB

## Abstract

Dry eye disease (DED) is the most common disease affecting vision and quality of life. PM_2.5_ was a potential risk of DED. Herein, we conducted animal exposure and cell-based studies to evaluate the pathogenic effect of PM_2.5_ exposure on the ocular surface and DED etiological mechanisms. C57 mice were exposed to filtered air and PM_2.5_ aerosol. We assessed health conditions and inflammation of the ocular surface by corneal fluorescein staining and immunohistochemistry. In parallel, cultured human corneal epithelial cells (HCETs) were treated with PM_2.5_, followed by characterization of cell viability, intracellular ATP level, mitochondrial activities, and expression level of DED relevant mRNA and proteins. In mice, PM_2.5_ exposure induced severe superficial punctate keratopathy and inflammation in their cornea. In HCETs, cell proliferation and ROS generation followed dose-response and time-dependent manner; meanwhile, mitochondrial ROS (mtROS) level increased and mitochondrial membrane potential (MMP) level decreased. Inflammation cascade was triggered even after short-term exposure. The reduction of ATP production was alleviated with Nrf2 overexpression, NF-κB P65 knockdown, or ROS clearance. Nrf2 overexpression and P65 knockdown reduced inflammatory reaction through decreasing expression of P65 and increasing of Nrf2, respectively. They partly alleviated changes of ROS/mtROS/MMP. This research proved that PM_2.5_ would cause DED-related inflammation reaction on corneal epithelial cells and further explored its mechanism: ROS from mitochondrial dysfunctions of corneal epithelial cells after PM_2.5_ exposure partly inhibited the expression of anti-inflammatory protein Nrf2 led the activation of inflammatory protein P65 and its downstream molecules, which finally caused inflammation reaction.

**Graphical abstract:**

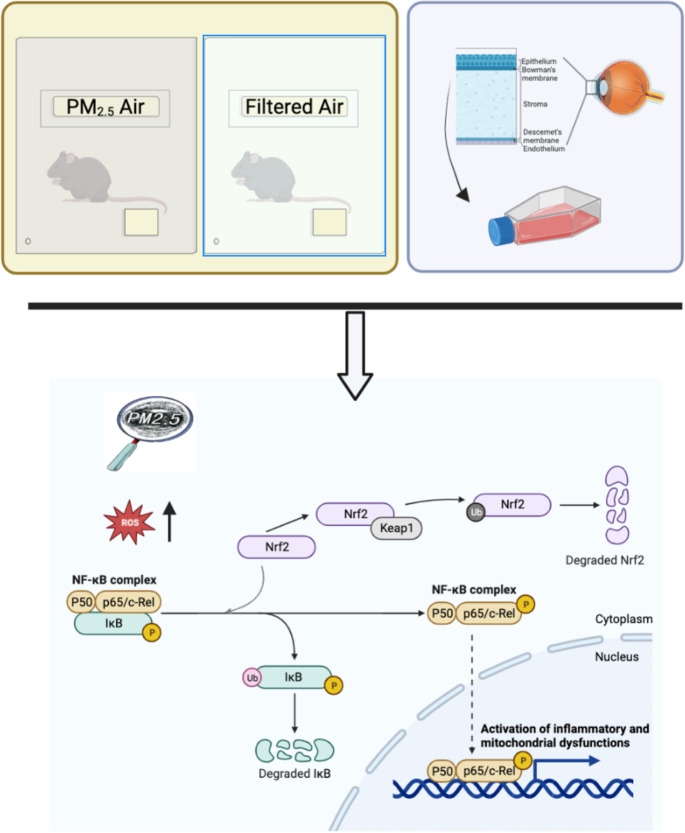

**Supplementary Information:**

The online version contains supplementary material available at 10.1007/s10565-023-09791-z.

## Introduction

Environmental pollution is the world’s largest cause of premature reversible death and disability. More than 70% of pollution-related illnesses are noninfectious chronic diseases, and nearly 92% of pollution-related deaths were from low- to middle-income countries (Landrigan et al. 2018). At present, particulate matter is the primary air pollutant in many cities and regions (Luo et al. 2020; Zhou et al. 2021). Fine particulate matter (PM) having a diameter of less than 2.5 μm (PM_2.5_) is discharged directly from a surface source formed in the atmosphere by a vapor/aqueous phase chemical reaction between precursor species (secondary particulate matter) (Akimoto 2003). PM_2.5_ is the world’s 5th largest risk factor for death, accounting for an estimated 7.6% of the global total in 2015 (Cohen et al. 2017). For every increase of 10 μg per m^3^ in PM_2.5_, there was an associated increase in all-cause mortality (Berger et al. 2017; Lipfert 2018). As a part of the human body surface, the cornea is in long-term contact with atmosphere including PM_2.5_. With prolonged exposure, the cornea is likely to be at risk of injury. Previous studies showed that tear stability and ocular surface integrity are climate-dependent (Mu et al. 2022).

Dry eye disease (DED) is a multifactorial disease where the homeostasis of ocular surface tear film was dysregulated and accompanied with ocular symptoms including tear film instability and hyperosmolarity, ocular surface inflammation and damage, and neurosensory abnormalities (Craig et al. 2017). In Asians, the prevalence of DED ranges from 10.4 to 37.9%, making it the most prevalent ocular surface disease (Gong et al. 2017; Titiyal et al. 2018; Um et al. 2014). In recent years, with the aggravation of air pollution, the prevalence of DED has increased year by year. The most frequently complaint of patients with DED is scratchy or sandy (foreign body). DED’s common signs and symptoms were not consistently correlated (Lemp et al. 2012). A lot of DED patients report ocular pain as a result of corneal damage disrupting of neural circuits and symptoms such as electric pain burning and tingling. The discomfort symptoms of DED are related to depression and anxiety (Al-Aqaba et al. 2019). Therefore, in this study, we put emphasis on the influence of PM_2.5_ on the cornea. Meanwhile, it has caused a huge economic burden to patients. In the USA, its annual medical and health care expenditure reaches $53.8 billion, and the DED’s overall burden would be 3.84 billion dollars per year from the US healthcare system and the average annual cost for a DED patient is $783 (Mertzanis et al. 2005). Comparative study showed that the related costs in the USA are roughly equivalent to those in other countries (McDonald et al. 2016).

The eyes are directly exposed to the air for a long time, so the impact of PM on the ocular surface is prominent. Previous epidemiologic studies showed significant associations between PM_2.5_ and ocular surface diseases such as DED and allergic conjunctivitis (Lu et al. 2021; Mo et al. 2019). Ocular surface inflammation is the main pathogenic factor of DED. Inflammatory cytokines, metalloproteinases, chemokines, and their receptors, are involved in the activation of immune cells, thus aggravating ocular inflammation. DED is related to nuclear factor κB (NF-κB) that is associated with the inflammatory cascade(De-Quan et al. 2013). The phosphorylation of both I*κ*Bα/pI*κ*Bα and p65/pp65 (NF-κB P65, RELA) is crucial for the inflammatory response. Nrf2 is a transcription factor that encourages expression of genes under stress and reduces electrophilic substances and reactive oxygen species (ROS) levels (Wei et al. 2020). Nrf2 is the main regulator to cellular redox homeostasis and Nrf2 signaling pathway plays a regulatory role in inflammation, especially in the inflammatory process (Barrera et al. 2021).

Our previous study also found that PM_2.5_ was a potential risk of DED(Yu et al. 2019). The aim of this study was to evaluate the effect of PM_2.5_ on the ocular surface and explore the pathogenesis of DED-related inflammation.

## Materials and methods

### PM_2.5_ aerosol preparation and experimental animals

The powder of PM_2.5_ was kindly provided by School of Public Health, Fudan University, Shanghai. Beijing Vital River Laboratory Animal Technology (Beijing, China) provided C57BL/6J male mice (64, 6 weeks old). In all animal experiments, the ARVO Statement for the Use of Animals in Ophthalmic and Vision Research was followed. Shanghai Tenth People’s Hospital’s ethical committee approved all animal experiments and consented to publish. All animals were raised in a filtered air room (PM free)/PM_2.5_ room for 3/7/10 weeks and were given free access to drinking water and common mice’s food (8 mice per group). They were exposed in FA/PM room for 8 h per day.

#### Fluorescent staining

Fluorescein sodium ophthalmic strips (Jingming new technological development Co, Tianjing, China) with normal saline were applied to the eye surface of mice. Meanwhile, slit lamp microscopy was used to observe damage to the eye surface using cobalt blue light.

The cornea surface was divided into four quadrants: upper quadrant, lower quadrant, nasal quadrant, and temporal quadrant. The score of each quadrant was presented separately, and then, a total score (16 points; Table 1) was added from each quadrant’s scores.

**Table 1:** The standard of corneal fluorescein staining

Score	Standard
0	No staining
1	Slight spot staining but less than 30 spots
2	Dot staining more than 30 spots, without diffuse staining
3	Severe diffuse staining, but no plaque
4	Plaque staining

#### Schirmer test

Under 1/3 of the conjunctiva, the phenol red cotton thread (PRT, Jingming new technological development Co) was bent 5 mm. We measured the red line's length after drawing it out for 20 seconds.

### Hematoxylin and eosin stain

Five-millimeter slices were cut from the paraffin-embedded retinal tissues. The sections were graded ethanol series and deparaffinized with xylene. After staining, incubating, and dehydrating, the tissues were mounted with neutral gum. A light microscope (Leica Microsystems, Wetzlar, Germany) was used to capture images.

### Immunohistochemistry

Corneal tissue sections (5 μm thick) were deparaffinized with xylene and an ethanol gradient. The tissues sections were blocked with goat serum after antigen retrieval. After that, the tissues sections were added with the respective primary antibodies in a volume of 100 μL and then incubated overnight. In the next day, biotin-labeled secondary antibody (Beyotime Biotechnology, Nanjing, Jiangsu, China) was added with the same volume. The tissues were counterstained by hematoxylin and were visualized with DAB for color appearance. Tissue images were captured by a light microscopy (Leica Microsystems).

### Cell culture and treatment

Adult human corneal epithelial cell line HCE-T cell was from MEISENCTCC company (#CTCC-002-0020, Hangzhou, Zhejiang, China). DMEM/F12 culture media (#30023.01, Thermo Fisher Scientific, Waltham, MA, USA) was added with 10% fetal bovine serum (FBS, Gibco, Carlsbad, CA, USA), 10 ng/ml human epidermal growth factor (hEGF; #91077C, Sigma-Aldrich, San Francisco, CA, USA), and 5 μg/ml insulin (INS; #E9644 Sigma-Aldrich). Cell culture environment was under an 5% CO_2_ atmosphere at 37 °C. The cell culture medium needs to be changed every day, and cells were seeded in 96-, 24-, 12-, or 6-well plates as needed. At 80% confluence, the cells were washed and treated with vehicle/tert-butylhydroquinone (tBHQ, 20μM; #T5364, TopScience, Shanghai, China)/Bay11-7082 (7.5 μM; #T1902, TopScience)/N-acetyl-LL-cysteine (NAC, 10 mM; #A9165, Sigma-Aldrich) for 6 h/24 h/3 h before exposure to PM_2.5_.

### RNA isolation and sequencing

Total RNA from HCETs was isolated using FastPure^®^ Cell/Tissue Total RNA Isolation Kit V2 (Vazyme Biotech Co., Nanjing, China) according to the manufacturer’s instructions. The libraries were established using VAHTS^®^ Universal V8 RNA-seq Library Prep Kit for Illumina (Vazyme). Related analyses were performed by OE Biotech Co.

### HCET transfection

HECTs were transfected with plasmids and siRNA using Lipofectamine2000 (#11668019, Thermo) for 4 h according to the manufacturer’s protocol before 24 h PM_2.5_ exposure. RT-qPCR and western blotting were used to evaluate the effect of transfection. Sequences of siRNA used are listed as follows: hRELA si-1 sense GGAGCACAGAUACCACCAATT; hRELA si-1 antisense UUGGUGGUAUCUGUGCUCCTC; hRELA si-2 sense CCUUUCUCAUCCCAUCUUUTT; hRELA si-2 antisense AAAGAUGGGAUGAGAAAGGAC; hRELA si-3 sense GGACAUAUGAGACCUUCAATT; and hRELA si-3 antisense UUGAAGGUCUCAUAUGUCCTT.

### Cell viability assay

Cell counting kit-8 (CCK-8 assay; #40203ES60, Yeasen, Shanghai, China) was used for cell viability. Procedures of related experiments were directed by the manufacturer’s instructions.

### The intracellular ATP level determination

ATP Assay Kit (#S0026, Beyotime) was used to determinate the intracellular ATP level. Procedures of related experiments were directed by the manufacturer’s instructions.

### Mitochondrially related fluorescence staining

HCETs were grown to 50% confluency and then incubated with each vehicle shown below before PM_2.5_ exposure. The 2′,7′-dichlorofluorescein diacetate (DCFH-DA, 10μM; #287810, Sigma-Aldrich)/MitoSox superoxide indicator (5 μM; #36008, Thermo Fisher Scientific)/tetramethylrhodamine ethyl ester perchlorate (TMRE, 200 nM; #T669, Sigma-Aldrich) were incubated with HECTs in serum-free medium for 30/40/10 min at 37°C with or without Hoecst (1 μM; #33342, Thermo Fisher Scientific). Fluorescence intensity was measured using a flow cytometer or a microplate reader (BioTek) or an inverted fluorescence microscope (Nikon Corporation) as needed.

### Immunofluorescence staining

HCETs were seeded and cultured in 35 mm confocal dishes with each vehicle. The cells were incubated with primary antibodies (all 1:800 dilution) overnight at 4 °C and fluorescein isothiocyanate (cy3/488)–conjugated secondary antibodies (all 1:500 dilution) for an hour. Nuclei were stained with DAPI (1:1,000 dilution; #D9542, Sigma-Aldrich) for 15 min. Confocal microscopy (LSM710; Carl Zeiss, Jena, Germany) was used to obtain the images.

### Western blotting

Protein extracted from the corneal tissues and cells was prepared by using RIPA (#P0013B, Beyotime) with a protease inhibitor (EMD Millipore, Billerica, MA, USA) and phosphatase inhibitors (50 mM NaF and 100μM Na_3_VO_4_; #G2007, Servicebio, Wuhan, Hubei, China) on ice for 30 min. A total of 30 μg protein was loaded on per channel of 10–12.5% polyacrylamide gels and after electrophoresis; they were transferred to polyvinylidene difluoride (Whatman, UK). After blocking, the membranes were incubated with primary antibodies against Nrf2 (1:500; #A11159, ABclonal, Wuhan, Hubei, China), NF-κB P65 (1:1000; #6956, Cell Signaling Technology), p-NF-κB pP65 (1:1000; #3033, Cell Signaling Technology), IKBα(1:1000; #T55026, Abmart, Shanghai, China), p-IKBα (1:2000; #T55572, Abmart), KEAP1 (1:1000; #8047, Cell Signaling Technology), IFNγ (1:200; #sc-8423, Santa Cruz, Dallas, TX, USA), HO1 (1:1000; #10701-1-AP, Proteintech, Wuhan, Hubei, China), SOD1 (1:500; #10269-1-AP, Proteintech), CAT (1:2000; #21260-1-AP, Proteintech), TNFα (1:500; #17590-1-AP, Proteintech), IL-1β (1:500; #AF7209, Beyotime Biotechnology), tubulin (1:5,000; #M30109, Abmart), and GAPDH (1:2000; #GB15001, Servicebio) at 4 °C overnight. After that, membranes incubated with secondary anti-rabbit/mouse antibodies (1:5,000; #926-32211/#926-32210, LI-COR Biosciences, Lincoln, NE, USA) for 1 h protected from light. Odyssey CX infrared laser imaging system (LI-COR Biosciences) was used to scan for pictures. The band intensity was estimated and analyzed by Image Studio Lite (Version 5.2.5) and ImageJ (Version 2.0.0-rc-43/1.50e).

### Quantitative PCR

EZ-press RNA purification Kit (Roseville, MN, USA) was used to extract total RNA. The primer sequences (Sangon Biotech, Shanghai, China) used are listed as fellow: human-Nrf2- F (5′-ATGTGGAGATCATTGAGCAGC-3′), human-Nrf2-R (5′-CCTGGTCCTGTGTAGCCATT-3′); human-NF-κB-F (5′-TCAGCGACGGAAAGAGTATGA-3′), human-NF-κB-R (5′-CCACTGGTTTCTGACTGGATGT-3′).

### Statistics analysis

GraphPad Prism 8 (Version:8.2.1) was used to perform statistical analyses and figures. All data was shown as the mean ± SD, and one-way ANOVA analysis and *t*-tests were performed to determine statistically significant. *P* < 0.05 means statistically significant.

## Results

### The influence of particulate matters 2.5 on ocular surface of C57/BL mice

**Fig. 1:**
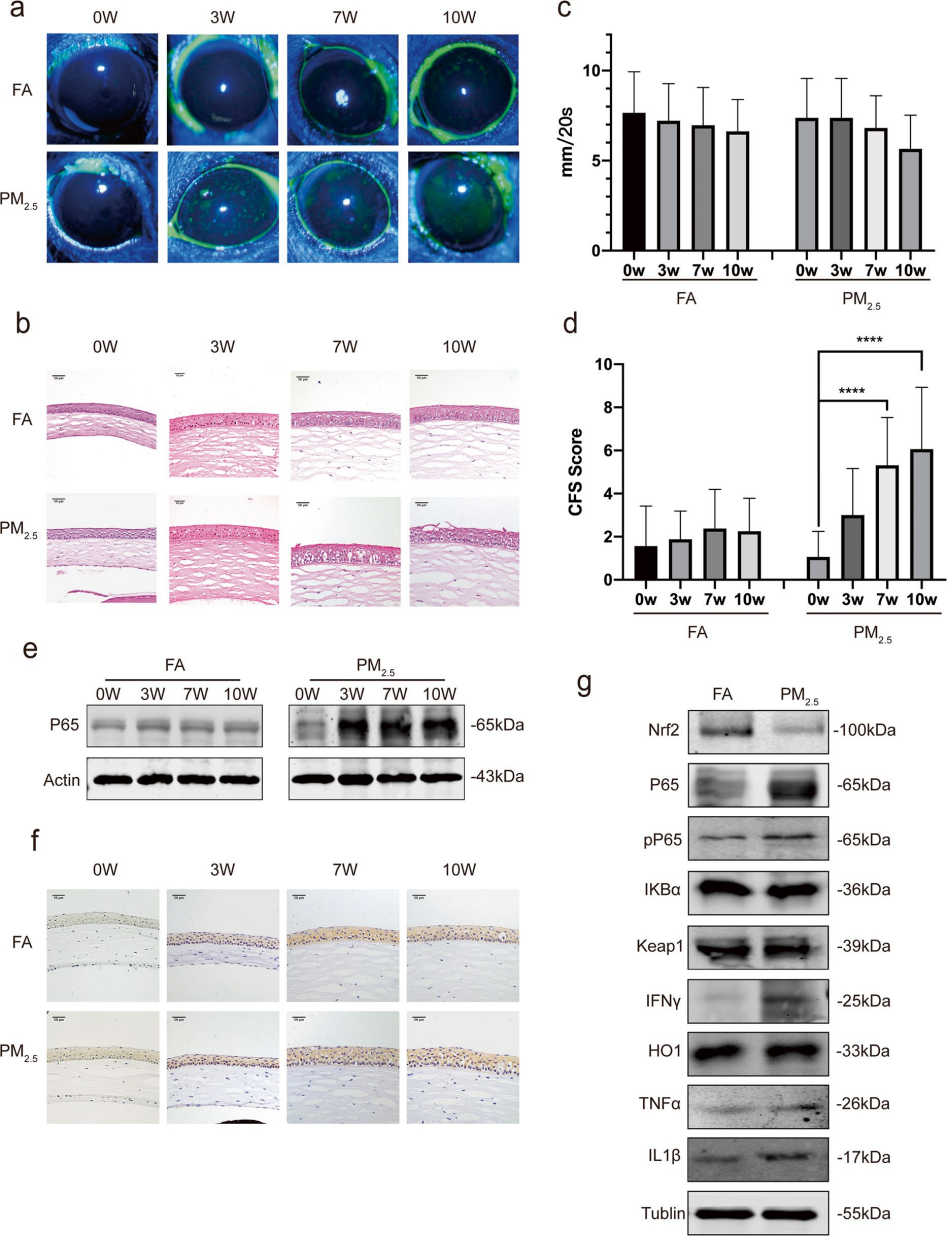
**a** Mice corneal staining pictures taken in 0, 3, 7, and 10 weeks, respectively; **b** hematoxylin and eosin stain of mice’s corneal in 0, 3, 7, and 10 weeks, respectively; **c** Schirmer test on mice (the phenol red cotton thread) in 0, 3, 7, and 10 weeks, respectively; **d** fluorescent staining scores on mice’s corneal epithelium in 0, 3, 7, and 10 weeks, respectively; **e** the results of WB on mice’s corneal in 0, 3, 7, and 10 weeks, respectively; **f** immunohistochemistry of IL-1β on mice’s corneal in 0, 3, 7, and 10 weeks, respectively; **g** the results of WB on mice’s corneal exposed for 10 weeks. Data represent mean ± SD of at least three independent experiments; *p < 0.05; **p < 0.01; ***p < 0.001; ****p < 0.0001, compared versus control

In our previous study, PM_2.5_ was one of the potential risk factors for prevalence of DED in human. To simulate the influence on the ocular surface of PM_2.5_, mice were exposed to filtered-air environment and PM_2.5_ environment randomly. Corneal fluorescein staining was performed to evaluate the severity of DED. Mice corneal staining pictures are shown in Fig. 1a from the first day up to 10 weeks. The histopathological alteration of corneal revealed detachment, swelling, and disorganization of corneal epithelium more frequently in PM-exposed eyes compared with FA-exposed eyes, which mean severe superficial punctate keratopathy happened (Fig. 1b). The increase of scores was related with an ophthalmic evaluation of superficial punctate keratopathy. The fluorescein staining scores (Fig. 1d) of PM-exposed mice were significantly increased compared with FA-exposed mice. Interestingly, no significant difference was observed in tear production between two groups whether on the first day or the 10 weeks (Fig. 1c) which revealed PM_2.5_ influences more on cornea than tear secretion function.

### Effect of PM_2.5_ on inflammation of cornea in C57/BL mice

To investigate the pathogenesis of the inflammation on ocular surface, we analyzed the protein expression of NF-κB P65 by WB (Fig. 1e). The results revealed that compared with FA-exposed group, NF-κB was significantly increased in PM-exposed group as exposed time prolonged. Immunohistochemical staining for inflammatory protein IL-1β showed increased expression of protein IL-1β in PM-exposed group (Fig. 1f). To further investigate the related activation of inflammation pathways, we performed WB results for specific antibodies (Fig. 1g). It revealed that the expression of protein Nrf2 and HO1, its downstream molecule, was decreased. And the expression of protein P65 and its downstream molecules (IL-1β and TNF-α) was increased. NF-κB P65 is silenced in the cytoplasm by an inhibitory protein, IkB, and one of the first genes becomes phosphorylated promoting ubiquitination and degradation following NF-kB P65 activation is IkBα. The Kelch-like ECH-related protein 1 (Keap1) targets Nrf2 and undergoes ubiquitination and degradation by proteasome, thereby inhibiting its transcriptional activity and antioxidant responses. The decreased of IkBα expression and increase of Keap1 expression were the future evidence that both activated protein NF-κB and inhibited protein Nrf2 induced inflammatory reaction.

### Changes of HCET function after PM_2.5_ exposed

**Fig. 2:**
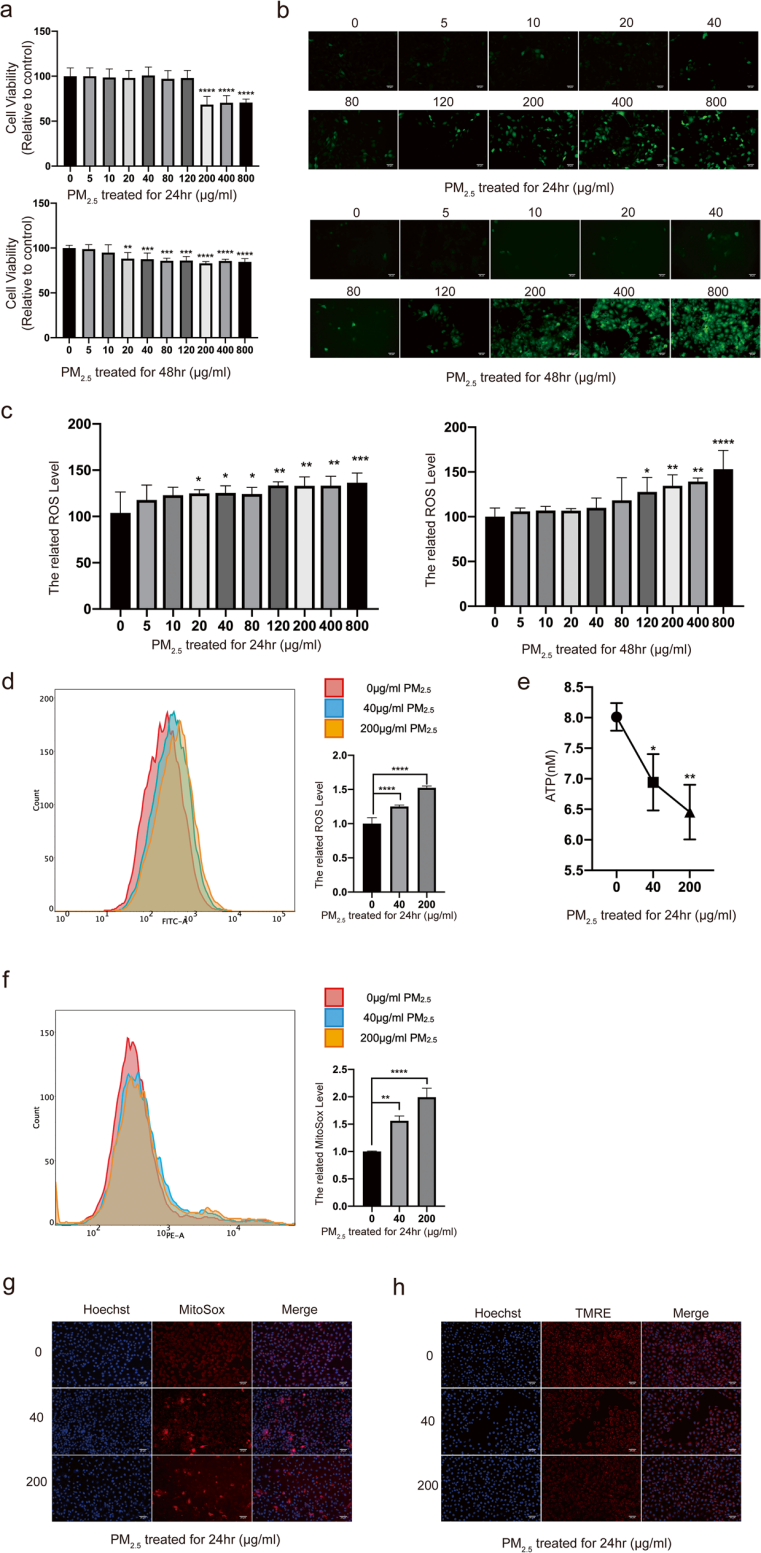
**a** The results of cell viability measured by a microplate reader which used cell counting kit-8; **b**, **c**, **d** the results of cell ROS production measured by an inverted fluorescence microscope (**b**), a microplate reader (**c**), and a flow cytometer (**d**) respectively which were treated with DCF-DA (10 μM) for 30 min after exposure; **e** The concentration of ATP after 24 h exposure; **f**, **g** the results of cell mtROS production measured by an inverted fluorescence microscope and a flow cytometer respectively which were treated with MitoSox (5 μM) for 40 min after exposure. Cell nucleuses were treated with Hoechst (1 μM) for 10 min; **h** the results of mitochondrial membrane potential measured by an inverted fluorescence microscope which were treated with TMRE (200 nM) for 10 min after exposure. Cell nucleuses were treated with Hoechst (1 μM) for 10 min. Data represent mean ± SD of at least three independent experiments; *p < 0.05; **p < 0.01; ***p < 0.001; ****p < 0.0001, compared versus control (PM_2.5_ 0 μg/ml)

Before CCK-8 analysis, HCETs were treated with different concentrations of PM_2.5_ for 24 h and 48 h. As shown in Fig. 2a, PM_2.5_ inhibited cell proliferation in a dose-dependent and time-dependent manner. Fluorescence intensity of ROS is shown in Fig. 2b and c. Fluorescence intensity increased with the increasing of PM_2.5_ concentration. Considering simulating a more realistic exposure environment, 40 mg/ml and 200 mg/ml were chosen as low-exposure and high-exposure concentrations. And flow cytometry showed the same result (Fig. 2d). There was statistical significance of ATP generation among different concentrations of PM_2.5_ (Fig. 1e), which showed that mitochondrial damage had already existed in 24-h treatment. To further study mitochondrial, fluorescence dye MitoSox (Fig. 2f and g) and TMRE (Fig. 2h) were used to stain the HCETs with different concentration exposures. We found that after PM_2.5_ exposure, MitoSox level, on behalf of mitochondrial ROS generation, increased and TMRE level, on behalf of mitochondrial membrane potential (MMP), decreased.

To better identify the functional pathways and important candidate genes which influenced by PM_2.5_, a comparative transcriptome analysis was performed by RNA-seq between HCETs with/without PM_2.5_ (200 μg/ml). Preprocessing result of data quality and result of data compared with reference genome are shown in Table S1 and Table S2. Gene expression level and correlation coefficient test showed that the biological reproducibility was good within sample group and the difference between groups was obvious (Table S3; Fig. S1). From this analysis, we identified 461 differentially expressed genes in these two groups. The distribution trends of DEGs in the pairwise comparisons are presented in the figures (Fig. S2–S5).

### Expression of inflammation related proteins in HCETs under PM_2.5_ treatment

**Fig. 3:**
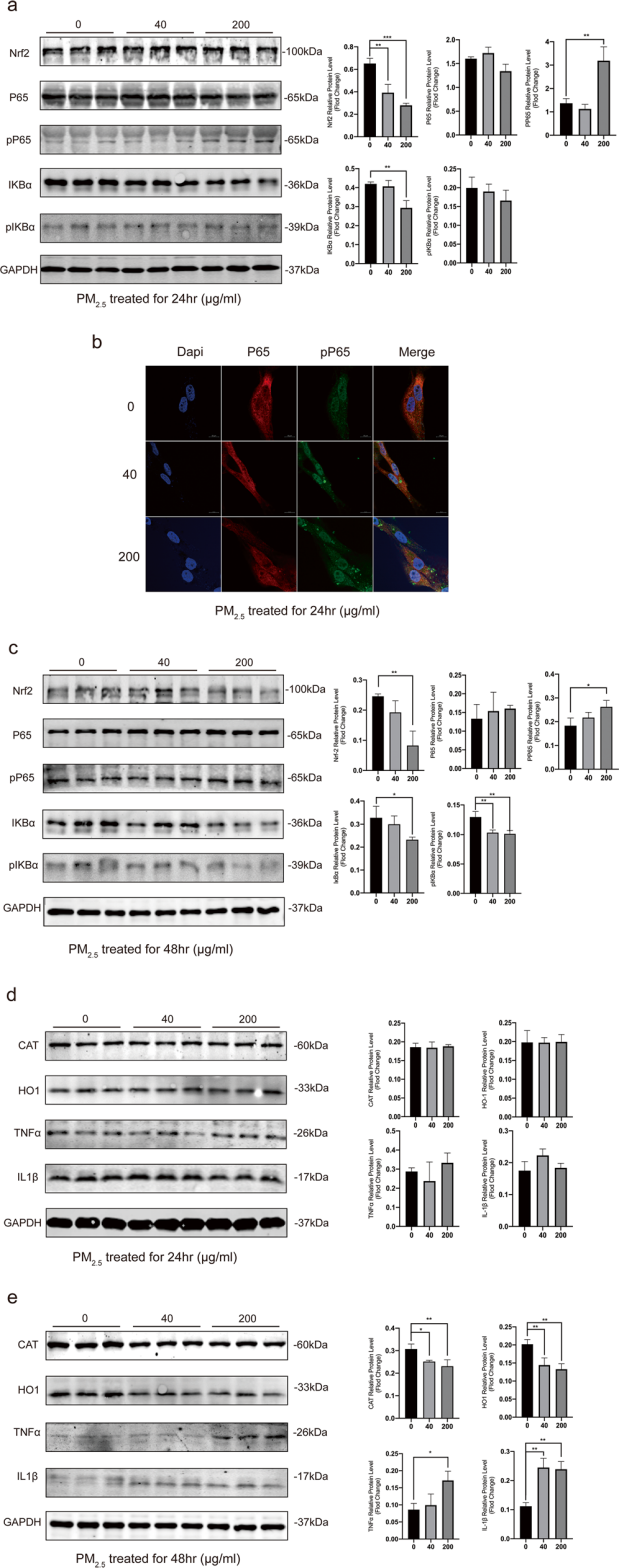
**a**, **c**, **d** The results of WB and gray value in HCETs’ proteins after 24 and 48 h exposure; **b** confocal pictures of p65 and pp65 protein immunofluorescence staining after 24 h exposure. Data represent mean ± SD of at least three independent experiments; *p < 0.05; **p < 0.01; ***p < 0.001; ****p < 0.0001, compared versus control (PM_2.5_ 0 μg/ml)

IkBα inhibits the NF-κB complex, and it is phosphorylated after stimulated on serine residues marking it for degradation by the ubiquitin pathway. Then, the NF-κB complex is phosphorylated and then translocates to the nucleus and activate transcription. As shown in Fig. 3a, for the first 24 h, the inflammation had been started. Although expression of protein p-IkBα did not change, expression of protein total IkBα decreased and the expression of protein pP65 increased, which revealed that protein P65 was activated. Interestingly, as an anti-inflammatory factor, the expression of protein Nrf2 should have increased, but it did not. The immunofluorescence results showed (Fig. 3b) that within 24 h, protein P65 was translocated to the nucleus and the expression of protein p-NF-κB pP65 in nucleus increased in a dose-dependent. When HCETs exposed in PM_2.5_ for 48 h (Fig. 3c), the expression of protein Nrf2 started to decrease and the expression of protein IkBα and p-IkBα continued to decrease while the expression of protein pP65 still decreased. Additionally, as was shown in Fig. 3d, there were no obvious changes in the expression of downstream proteins in 24 h, while there were obvious changes in its downstream proteins in 48 h.

### Expression of Nrf2 and NF-κB P65 proteins in HCETs influenced by PM_2.5_ exposure via ROS production

**Fig. 4:**
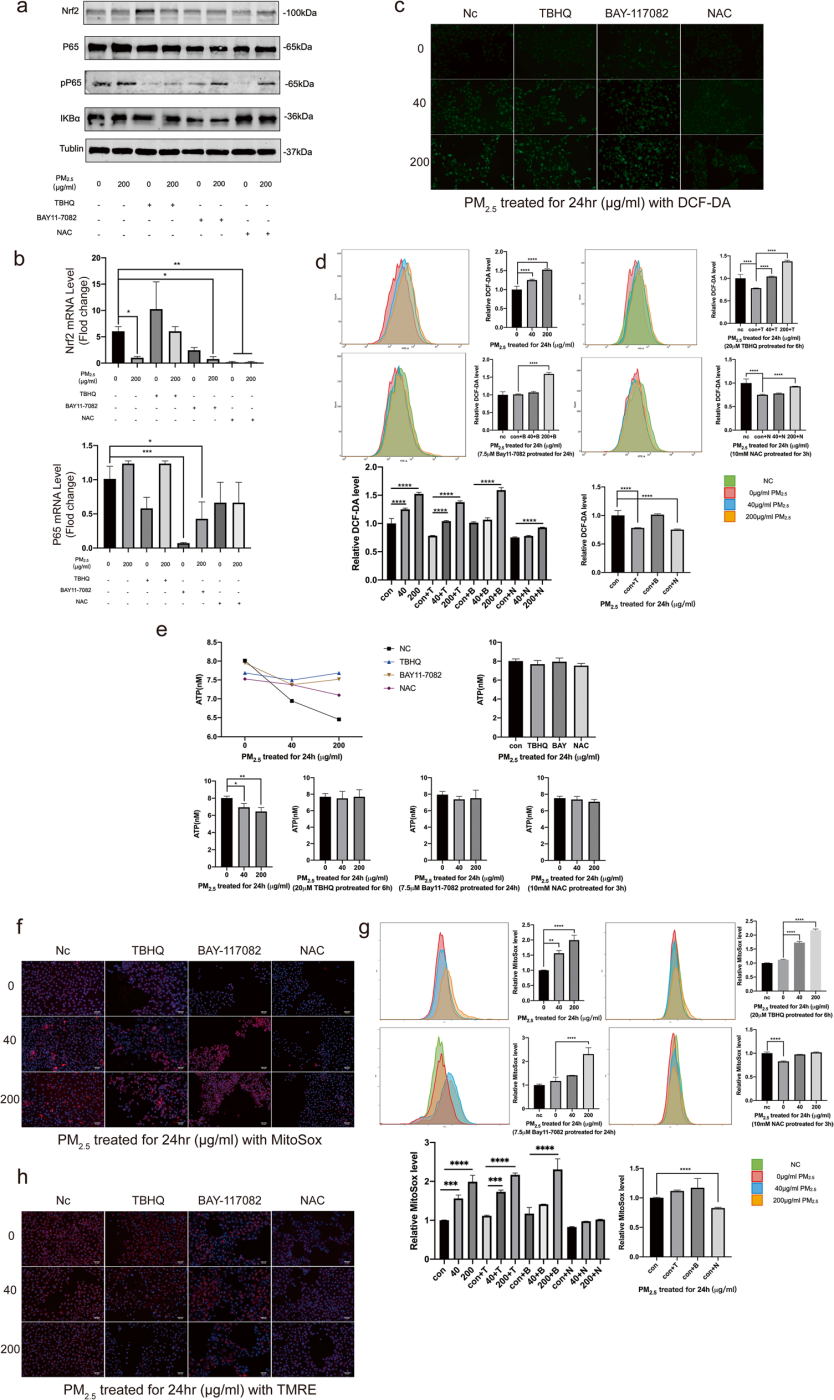
**a**, **b** The results of WB and qPCR in HCETs’ proteins after 24 h exposure; **c**, **d** the results of cell ROS production measured by an inverted fluorescence microscope and a flow cytometer respectively which were treated with DCF-DA (10 μM) for 30 min after exposure; **e** the concentration of ATP after 24 h exposure; **f**, **g** the results of cell mtROS production measured by an inverted fluorescence microscope and a flow cytometer respectively which were treated with MitoSox (5 μM) for 40 min after exposure. Cell nucleuses were treated with Hoechst (1 μM) for 10 min; (h) the results of mitochondrial membrane potential measured by an inverted fluorescence microscope which were treated with TMRE (200 nM) for 10 min after exposure. Cell nucleuses were treated with Hoechst (1 μM) for 10 min. The cells were treated with vehicle/TBHQ (20 μM, 6 h)/Bay11-7082 (7.5 μM, 24 h)/NAC (10 mM, 3 h) before exposure to PM_2.5_. Data represent mean ± SD of at least three independent experiments; *p < 0.05; **p < 0.01; ***p < 0.001; ****p < 0.0001, compared versus control

From the previous experiments, we found that the expression of Nrf2 and NF-κB P65 proteins in HCETs had been influenced after 24 h exposure so that the further exploration was all in 24 h. TBHQ is a Nrf2 activator, and BAY 11-7082 is a NF-κB P65 inhibitor. Acetylcysteine is the N-acetyl derivative of cysteine (NAC). It is used as the ROS scavenger. Treated with tBHQ, Bay11-7082, or NAC, all decreased the expression of inflammatory protein (Fig. 4a). The results of qPCR also show the same changes (Fig. 4b). PM_2.5_ exposure reduced ATP production; however, there were no significant changes on ATP production after tBHQ, Bay11-7082, or NAC treatment (Fig. 4e) which meant these three treatments would reduce mitochondrial damage. This may be related to the remission of inflammatory response. Although tBHQ decreased total ROS production without PM_2.5_ exposure, there were no influences on total ROS or mtROS production with the treatments of tBHQ or Bay11-7082 after PM_2.5_ exposure (Fig. 4c, d, f, g; Fig. S6a). The results of MMP show the same changes (Fig. 4h; Fig. S6b). It showed the potential relationship among ROS, P65, and Nrf2.

**Fig. 5:**
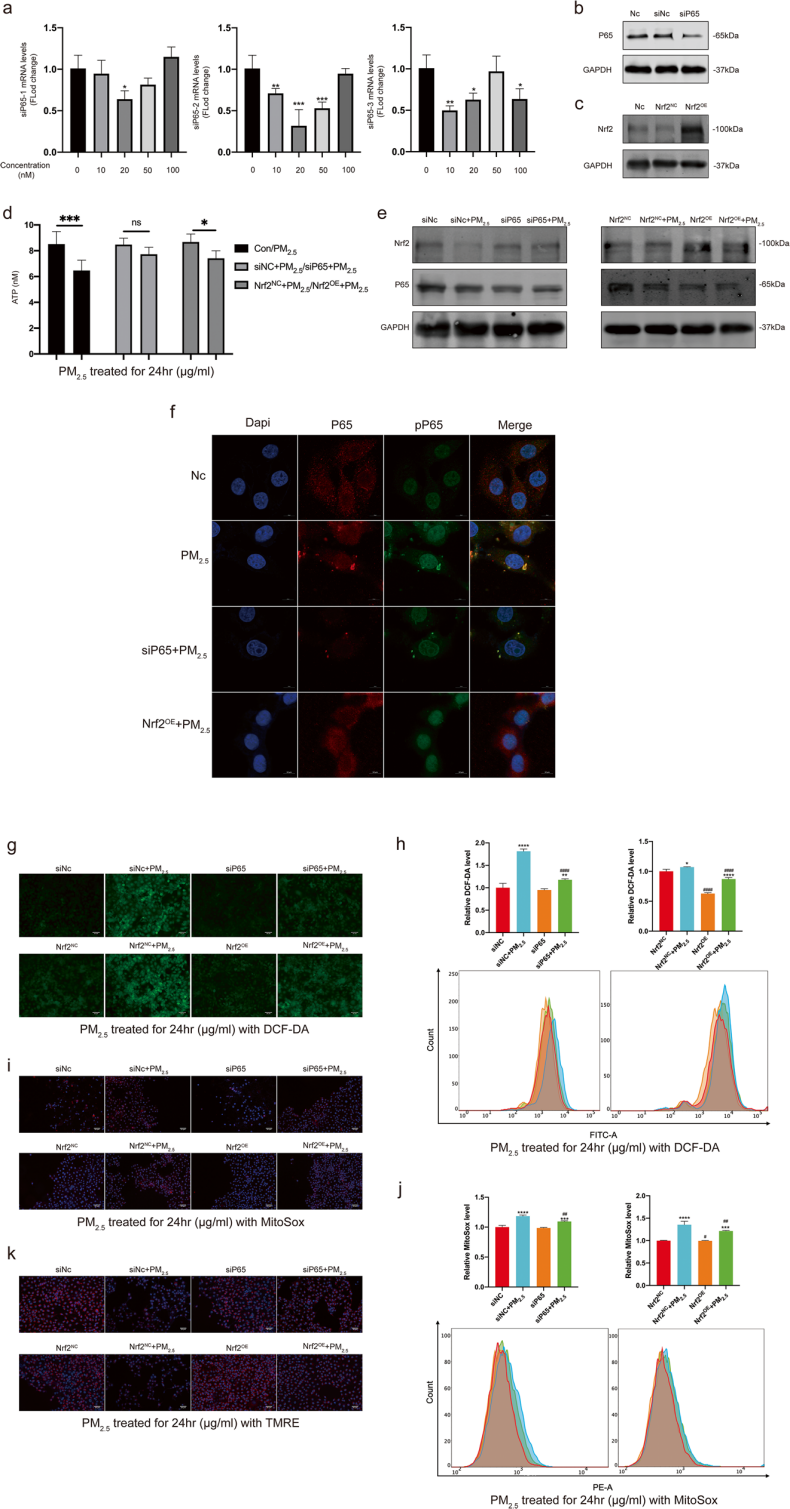
**a** The results of qPCR in 3 siP65 groups with different concentrations (0/10/20/50/100 nM); **b**, **c** The WB results of verification on siNC/siP65 (siP65-2, 20 nM) and Nrf2^NC^/Nrf2^OE^; **d** the concentration of ATP after 24 h exposure (200 μg/ml); **e** the results of WB a in HCETs’ proteins after 24 h exposure (200 μg/ml); **f** confocal pictures of p65 and pp65 protein immunofluorescence staining after 24 h exposure (200 μg/ml); **g**, **h** the results of cell ROS production measured by an inverted fluorescence microscope and a flow cytometer respectively which were treated with DCF-DA (10 μM) for 30 min after exposure (200 μg/ml); **i**, **j** the results of cell mtROS production measured by an inverted fluorescence microscope and a flow cytometer respectively which were treated with MitoSox (5 μM) for 40 min after exposure (200 μg/ml). Cell nucleuses were treated with Hoechst (1 μM) for 10 min; **k** the results of mitochondrial membrane potential measured by an inverted fluorescence microscope which were treated with TMRE (200 nM) for 10 min after exposure (200 μg/ml). Cell nucleuses were treated with Hoechst (1 μM) for 10 min. Data represent mean ± SD of at least three independent experiments; ^*/#^*p* < 0.05; ^**/##^*p* < 0.01; ^***/###^*p* < 0.001; ^****/####^*p* < 0.0001, compared versus control. *Compared with PM_2.5_-free group with the same knockdown/overexpress way. ^#^Knockdown/overexpress groups compared with their negative controls in the same concentration of PM_2.5_

For further exploration, the NF-κB P65 knockdown and Nrf2 overexpression models of HCETs were constructed. Three groups of siP65 with different concentrations (10/20/50/100 nM) were used to transfect HCETs (Fig. 5a), and finally, we chose siP65-2 in 20 nM for further verification (Fig. 5b). Meanwhile, we also verified the transient transfection of plasmids (Fig. 5c). NF-κB P65 knockdown and Nrf2 overexpression alleviated the reduction of ATP production (Fig. 5d), which mean mitochondrial damage was alleviated. Meanwhile, the result of Western blotting and confocal microscopy (Fig. 5e, f; Fig. S7a) showed NF-κB P65 knockdown and Nrf2 overexpression increased the expression of Nrf2 and decreased the expression of NF-κB P65 then restrained protein NF-κB P65 from translocating to the nucleus. To gain further insight into mitochondrial damage, ROS (Fig. 5g and h) and mtROS (Fig. 5i, j; Fig. S7b) were observed by flow cytometry and fluorescence microscopy and we found the reduction of ROS and mtROS production after NF-κB P65 knockdown and Nrf2 overexpression. The results of MMP show that NF-κB P65 knockdown and Nrf2 overexpression alleviated the reduction of MMP (Fig. 5k; Fig. S7c).

## Discussion

The classification by Asia Dry Eye Society was divided into three categories in 2020: increased evaporation (EDE), aqueous deficiency (ADDE), and decreased wettability. Every type of DED can be presented with related abnormalities in the ocular surface epithelium (Labetoulle et al. 2022). Tear hyperosmolarity is the core mechanism of DED, which is a vicious circle (Rolando and Zierhut 2001). Quantitative or qualitative defects in tears generally lead to wetting defects, tear film instability and hypertonic pressure, increased friction, and chronic mechanical stimulation of ocular surface. This triggers a series of inflammatory events and surface damage, which is characteristic of the disease (Bron et al. 2017). PM has robust proinflammatory effects because the insoluble nanoparticles act as immunostimulants. PM_2.5_ can trigger inflammation with systemic effects through the release of proinflammatory cytokines, increase of ROS, and impairment of the antioxidant system. In response, oxidative stress further triggers inflammatory and mutagenic responses (Offer et al. 2022). Mitochondria and endocytoplasmic reticulum (ER) may be susceptible to PM (including its components). Some ultrastructural changes happened in mitochondria and ER. Mitochondrial permeability transition pore opening, mitophagy, elimination in mitochondrial potential, reduction in ATP production, decline in mitochondrial DNA (mtDNA) copy number, and increased dynamic signaling in mitochondria happened after PM exposure (Piao et al. 2018; Wang et al. 2019; Zhang et al. 2018; Zhu et al. 2022). In this research, we found that PM_2.5_ would hurt corneal epithelium while the effect on tear fluid secretion was limited. The toxic reaction of PM_2.5_ was mainly manifested in the inflammatory reaction on the ocular surface.

ROS encompasses oxygen-free radicals and nonradical oxidants. According to normal fluctuation of energy load, it is safe to the maintenance of function of the biological system with the productions of ROS and its levels in mitochondria, cells, and tissues. However, with ROS production overwhelming, when it is not compensated by endogenous antioxidants for their scavenging, it will lead the rise of ROS called oxidative stress (Zorov et al. 2014). Excessive ROS could be produced by PM_2.5_ exposure, and antioxidant enzyme activities could be reduced, which results in oxidative stress in cells (Zhao et al. 2019). The formation of ROS primarily occurs in the endoplasmic reticulum (ER) and mitochondria of eukaryotic cells (Zhao et al. 2019). Mitochondria are the main parts of aerobic respiration to provide energy and the main source of intracellular ROS (Chouchani et al. 2014). Oxidative damage led by mitochondrial ROS (mtROS) production has been associated with aging (Morais et al. 2014). The production of mtROS is a tightly and continuous regulated process, which required for the regulation of many life activities (Figueira et al. 2013). A precise control system was used to balance mtROS’s elimination and production. Oxidative stress damage could be alleviated by mtROS scavenging system. The protective enzyme system can efficiently eliminate ROS including glutathione peroxidases (GPXs), catalase (CAT), peroxiredoxins (PRXs), and superoxide dismutase (SOD)(Liguori et al. 2018). The overtopping intracellular ROS concentration makes the antioxidant defense system equipped with mitochondria which could reduce the cytotoxicity. O_2_^−^ can be efficiently changed to H_2_O_2_ by SOD, and then CAT, which mainly exists in the cells’ peroxisome and promotes the decomposition of H_2_O_2_ into O_2_ and H_2_O (Winterbourn and Hampton 2008). High mtROS levels and digestion will decrease MMP, and then, MitoSox may underestimate mtROS levels (Yang et al. 2021). Thus, the flow cytometer result of MitoSox in this research was possibly more obvious than physical truth.

As the primary transcription factor in cellular defense, Nrf2 acts as the major inspector for reduction-oxidation status and detoxification (Ahmed et al. 2016). Nrf2 preserves mitochondrial integrity and regulates MMP and the accessibility of substrates for mitochondrial respiration and ATP synthesis (Agyeman et al. 2012; Gough and Cotter 2011). Furthermore, NADPH levels reduced because of lack of Nrf2, which makes the cells change into an oxidized state, leading to inflammation and death (Morgan et al. 2013). Under physiological conditions, generally, Nrf2 stays in the cytoplasm and connected with Keap1(Nrf2 negative regulator) (Bhakkiyalakshmi et al. 2015). When the adequate activity and oxidant species of antioxidant proteins are balance, Nrf2 is suppressed in the cytoplasm (Piantadosi et al. 2008). However, after encountered oxidative stress including mtROS, Nrf2 is isolated from the Nrf2-Keap1 complex and then translocates to the nucleus to sustain cellular redox homeostasis (Agyeman et al. 2012; Li et al. 2019). Previous study showed that decrease of Nrf2 led the increase of oxidative damage and some pathophysiological disorders (Tebay et al. 2015). NF-κB transcription factor family, which upregulates the expression of genes underpinning a broad spectrum of biological processes(Zhang et al. 2017), is composed of NFKB1 (P105/P50),RELA (P65), NFKB2(P100/P52), RELB, and c-REL. RELA, c-REL, and RELB contain DNA-binding and transactivation domains. NF-κB family members form homodimers and heterodimers to induce or inhibit transcription, in which p65/p50 heterodimer typically regulates gene expression of IL-1 (Oeckinghaus and Ghosh 2009). In the classic NF-κB pathway, the phosphorylation of both I*κ*Bα (pI*κ*Bα) and p65 (pP65) is crucial. PI*κ*Bα is essential for the induction of degradation of I*κ*B*α*, and pP65 regulates its transcriptional activity. IκBα is phosphorylated by IKKβ, and then, pIκBα becomes ubiquitinated, followed by proteasomal degradation. The p65 subunit of NF-κB released from IκBα is subsequently phosphorylated by IKKβ or other kinases and moves into the nucleus (Karin and Ben-Neriah 2000). Activated NF-κB P65 transactivates the expression of a large array of inflammatory genes, including chemokines and cytokines such as TNF-α and IL-1β (Wang et al. 2013).

In this study, oxidative stress like ROS should have activated Nrf2 but PM_2.5_ decreased Nrf2, thereby inhibiting its downstream regulations, including CAT and HO-1 enhance that is maybe one of the reasons that inflammation (increase of ROS, dysfunction of mitochondria, and activation of NF-κB P65) happened on ocular surface. When ROS scavenged, Nrf2 decreased as it was supposed to be. P66shc is an adaptor protein that promotes oxidative stress which resides in the cytosol. Under oxidative stress, p66shc could be translocated in the mitochondria in a PKCβ-dependent manner, serving as an important source of reactive oxygen species (Pinton et al. 2007). P66shc-generated ROS activates NF-κB P65, thereby amplifying inflammation and oxidative stress (Menini et al. 2007). That is maybe another reason that increase of ROS, dysfunction of mitochondria, and activation of NF-κB P65 happened after PM_2.5_ exposure while the expression of NF-κB P65 decreased after ROS scavenged.

## Conclusions

This research proved that PM_2.5_ would cause DED-related inflammation reaction on corneal epithelial cells and further explored its mechanism: ROS from mitochondrial dysfunctions of corneal epithelial cells after PM_2.5_ exposure inhibited the expression of anti-inflammatory protein Nrf2 led the activation of inflammatory protein NF-κB P65 and its downstream molecules, which finally caused inflammation reaction.

### Supplementary Information

Fig. S1 (a) Gene expression in each sample; (b-e) Gene expression level including box-whisker plots (b), violin plots (c), density distribution curve (d), and stacked histogram (e); (f-i) Results of correlation test including heatmap (f), cluster analysis (g), and Principal Component Analysis (PCA) (h, i); (j-m) Quality Control including per base quality (j), per base sequence content (j), randomness evaluation of sequencing (i), and enrichment analysis of Reads in different elements (m). (PNG 1611 kb)

High Resolution Image (TIF 422262 kb)

Fig. S2 Analysis of differentially expressed genes (DEGs). (a) Statistical histogram of DEGs; (b) Cluster diagram of DEGs; (c) Radar map of DEGs; (d) Volcano plots of DEGs. DEGs were defined using the following criteria: FC, fold-change <0.5 or >2 and P < 0.05. (PNG 761 kb)

High Resolution Image (TIF 117744 kb)

Fig. S3 GO enrichment analysis of DEGs. (a) GO enrichment analysis in Level 2; (b) Top 30 genes from GO enrichment analysis; (c) Chord diagrams of GO enrichment analysis; (d) Circos plots of GO enrichment analysis; (e) Histograms of distribution of DEGs and total genes at GO level2; (f) Histograms of distribution of upregulated DEGs and downregulated DEGs at GO level2. DEGs were defined using the following criteria: FC, fold-change <0.5 or >2 and P < 0.05. (PNG 2686 kb)

High Resolution Image (TIF 289660 kb)

Fig. S4 KEGG enrichment analysis of DEGs. (a) Bubble charts of top 20 genes from KEGG enrichment analysis; (b) Chord diagrams of KEGG enrichment analysis; (c) Circos plots of KEGG enrichment analysis; (d) KEGG enrichment analysis of pathway classification; (e) Histogram of distribution of DEGs and total genes; (f) Histogram of distribution of upregulated DEGs and downregulated DEGs. DEGs were defined using the following criteria: FC, fold-change <0.5 or >2 and P < 0.05. (PNG 2940 kb)

High Resolution Image (TIF 217906 kb)

Fig. S5 Reactome and WikiPathways enrichment analysis of DEGs. (a) Bubble charts of top 20 genes from Reactome enrichment analysis; (b) Chord diagrams of top 10 classifications from Reactome enrichment analysis; (c) Bubble charts of top 20 genes from WikiPathways enrichment analysis; (d) Chord diagrams of top 10 classifications from WikiPathways enrichment analysis. (PNG 3129 kb)

High Resolution Image (TIF 181058 kb)

Fig. S6 (a) The results of cell mtROS production measured by an inverted fluorescence microscope and a flow cytometer respectively which were treated with MitoSox (5μM) for 40 minutes after exposure (200μg/ml). Cell nucleuses were treated with Hoechst (1μM) for 10 minutes; (b) The results of mitochondrial membrane potential measured by an inverted fluorescence microscope which were treated with TMRE (200nM) for 10 minutes after exposure (200μg/ml). Cell nucleuses were treated with Hoechst (1μM) for 10 minutes. (PNG 3310 kb)

High Resolution Image (TIF 129544 kb)

Fig. S7 (a) Con-focal pictures of p65 and pp65 proteins immunofluorescence staining after 24hr exposure (200μg/ml); (b) The results of cell mtROS production measured by an inverted fluorescence microscope and a flow cytometer respectively which were treated with MitoSox (5μM) for 40 minutes after exposure (200μg/ml). Cell nucleuses were treated with Hoechst (1μM) for 10 minutes; (c) The results of mitochondrial membrane potential measured by an inverted fluorescence microscope which were treated with TMRE (200nM) for 10 minutes after exposure (200μg/ml). Cell nucleuses were treated with Hoechst (1μM) for 10 minutes. (PNG 3836 kb)

High Resolution Image (TIF 34454 kb)

Table S1 (XLSX 10.1 kb)

Table S2 (XLSX 10.3 kb)

Table S3 (XLSX 10.6 kb)

## Data Availability

The datasets used or analyzed during the current study are available from the corresponding author on reasonable request.
