# Supplementary figures and images for: PM2.5 exposure increases dry eye disease risks through corneal epithelial inflammation and mitochondrial dysfunctions
Source: Cell Biol Toxicol. 2023 Feb 14;39(6):2615–30. doi: 10.1007/s10565-023-09791-z (PMC10693534; doi:10.1007/s10565-023-09791-z)

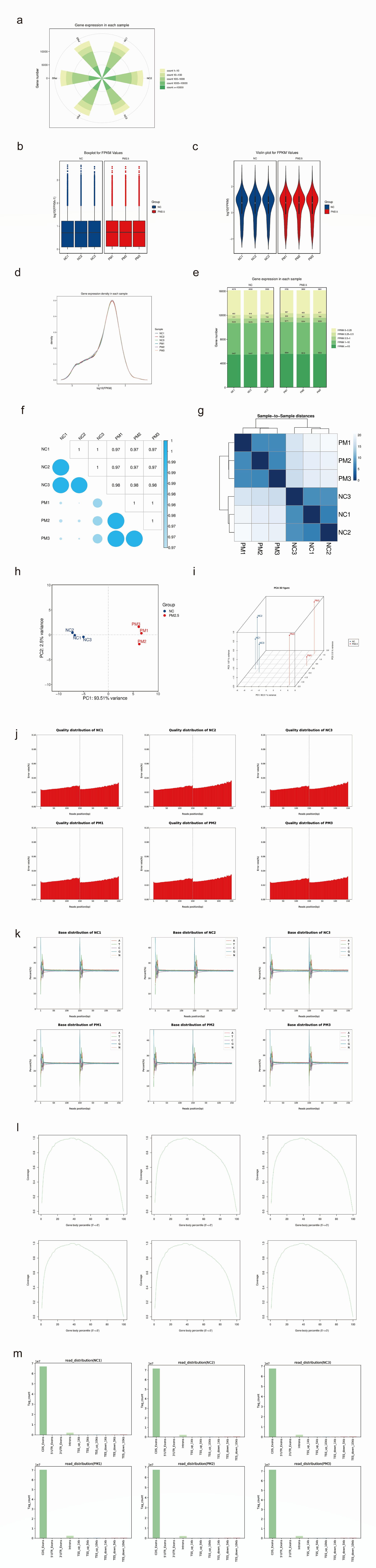

Supplement: Supplementary file 1 — (a) Gene expression in each sample; (b-e) Gene expression level including box-whisker plots (b), violin plots (c), density distribution curve (d), and stacked histogram (e); (f-i) Results of correlation test including heatmap (f), cluster analysis (g), and Principal Component Analysis (PCA) (h, i); (j-m) Quality Control including per base quality (j), per base sequence content (j), randomness evaluation of sequencing (i), and enrichment analysis of Reads in different elements (m). (PNG 1611 kb) [file 10565_2023_9791_Fig6_ESM.png]

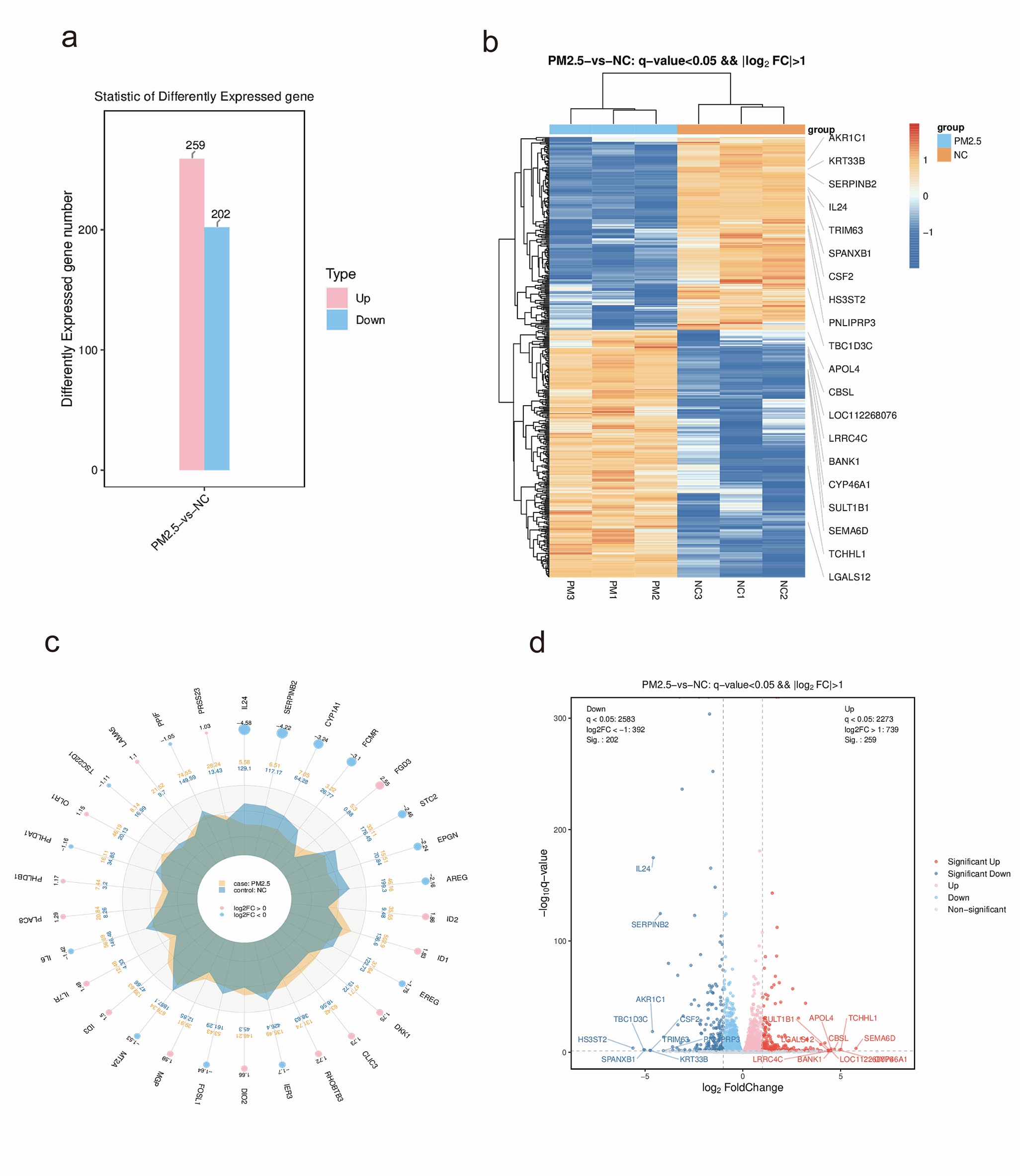

Supplement: Supplementary file 3 — Analysis of differentially expressed genes (DEGs). (a) Statistical histogram of DEGs; (b) Cluster diagram of DEGs; (c) Radar map of DEGs; (d) Volcano plots of DEGs. DEGs were defined using the following criteria: FC, fold-change <0.5 or >2 and P < 0.05. (PNG 761 kb) [file 10565_2023_9791_Fig7_ESM.png]

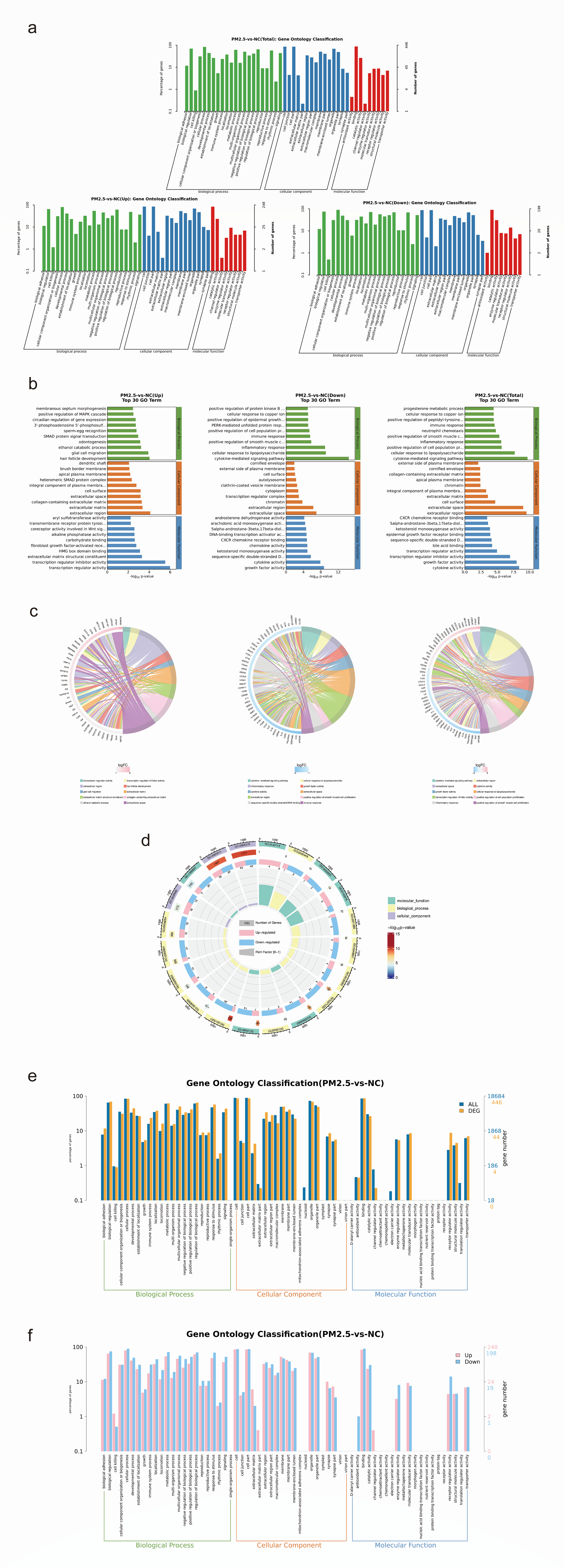

Supplement: Supplementary file 5 — GO enrichment analysis of DEGs. (a) GO enrichment analysis in Level 2; (b) Top 30 genes from GO enrichment analysis; (c) Chord diagrams of GO enrichment analysis; (d) Circos plots of GO enrichment analysis; (e) Histograms of distribution of DEGs and total genes at GO level2; (f) Histograms of distribution of upregulated DEGs and downregulated DEGs at GO level2. DEGs were defined using the following criteria: FC, fold-change <0.5 or >2 and P < 0.05. (PNG 2686 kb) [file 10565_2023_9791_Fig8_ESM.png]

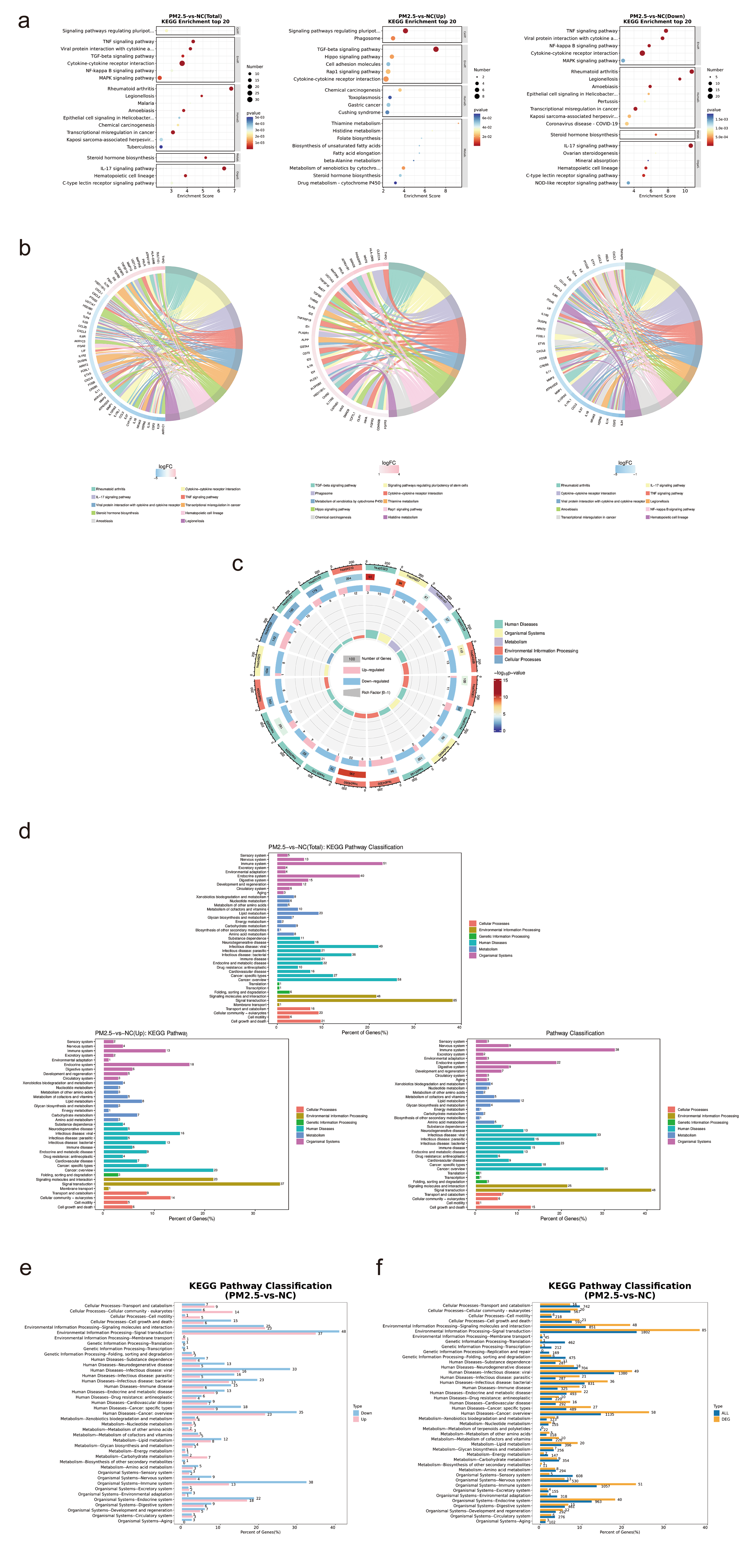

Supplement: Supplementary file 7 — KEGG enrichment analysis of DEGs. (a) Bubble charts of top 20 genes from KEGG enrichment analysis; (b) Chord diagrams of KEGG enrichment analysis; (c) Circos plots of KEGG enrichment analysis; (d) KEGG enrichment analysis of pathway classification; (e) Histogram of distribution of DEGs and total genes; (f) Histogram of distribution of upregulated DEGs and downregulated DEGs. DEGs were defined using the following criteria: FC, fold-change <0.5 or >2 and P < 0.05. (PNG 2940 kb) [file 10565_2023_9791_Fig9_ESM.png]

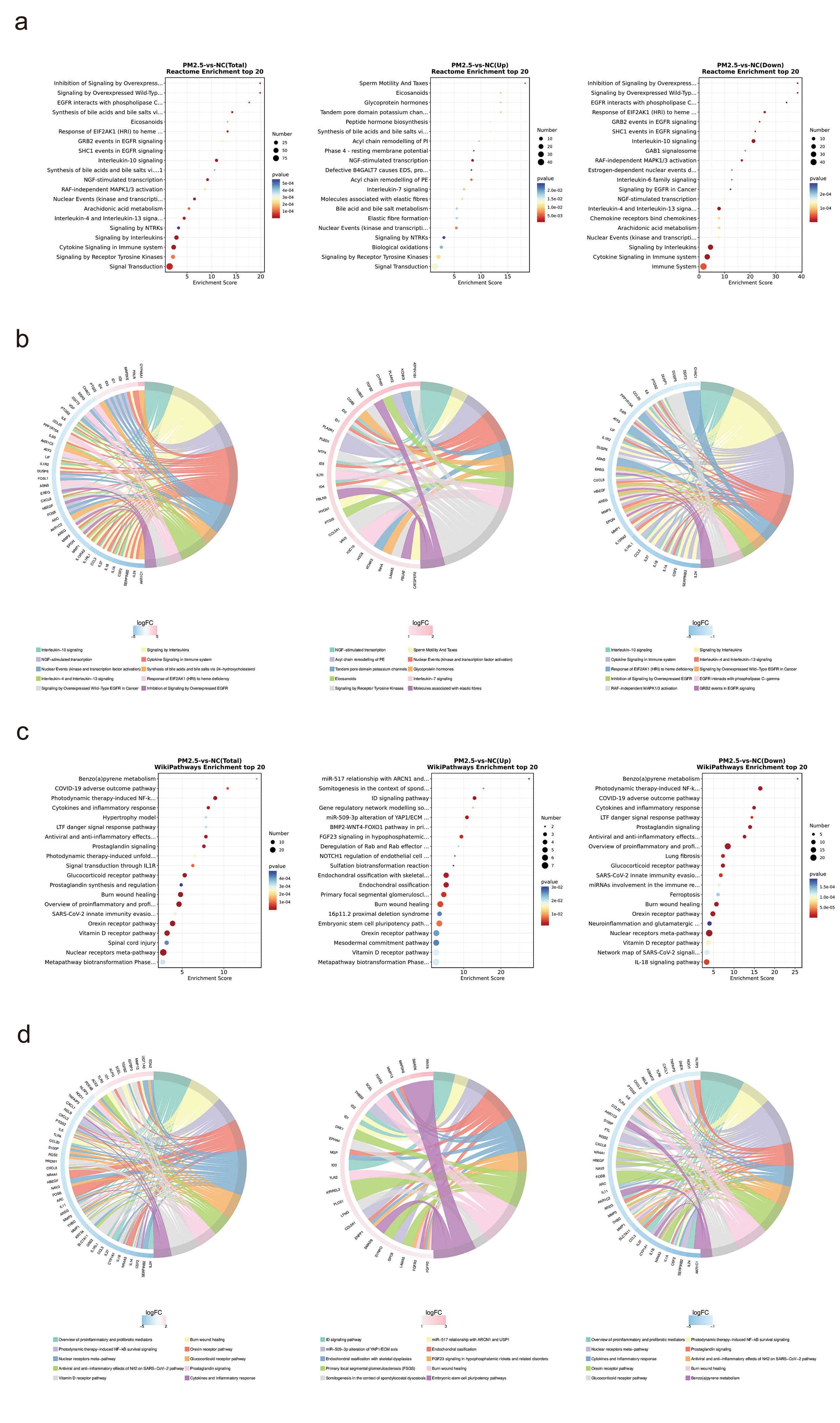

Supplement: Supplementary file 9 — Reactome and WikiPathways enrichment analysis of DEGs. (a) Bubble charts of top 20 genes from Reactome enrichment analysis; (b) Chord diagrams of top 10 classifications from Reactome enrichment analysis; (c) Bubble charts of top 20 genes from WikiPathways enrichment analysis; (d) Chord diagrams of top 10 classifications from WikiPathways enrichment analysis. (PNG 3129 kb) [file 10565_2023_9791_Fig10_ESM.png]

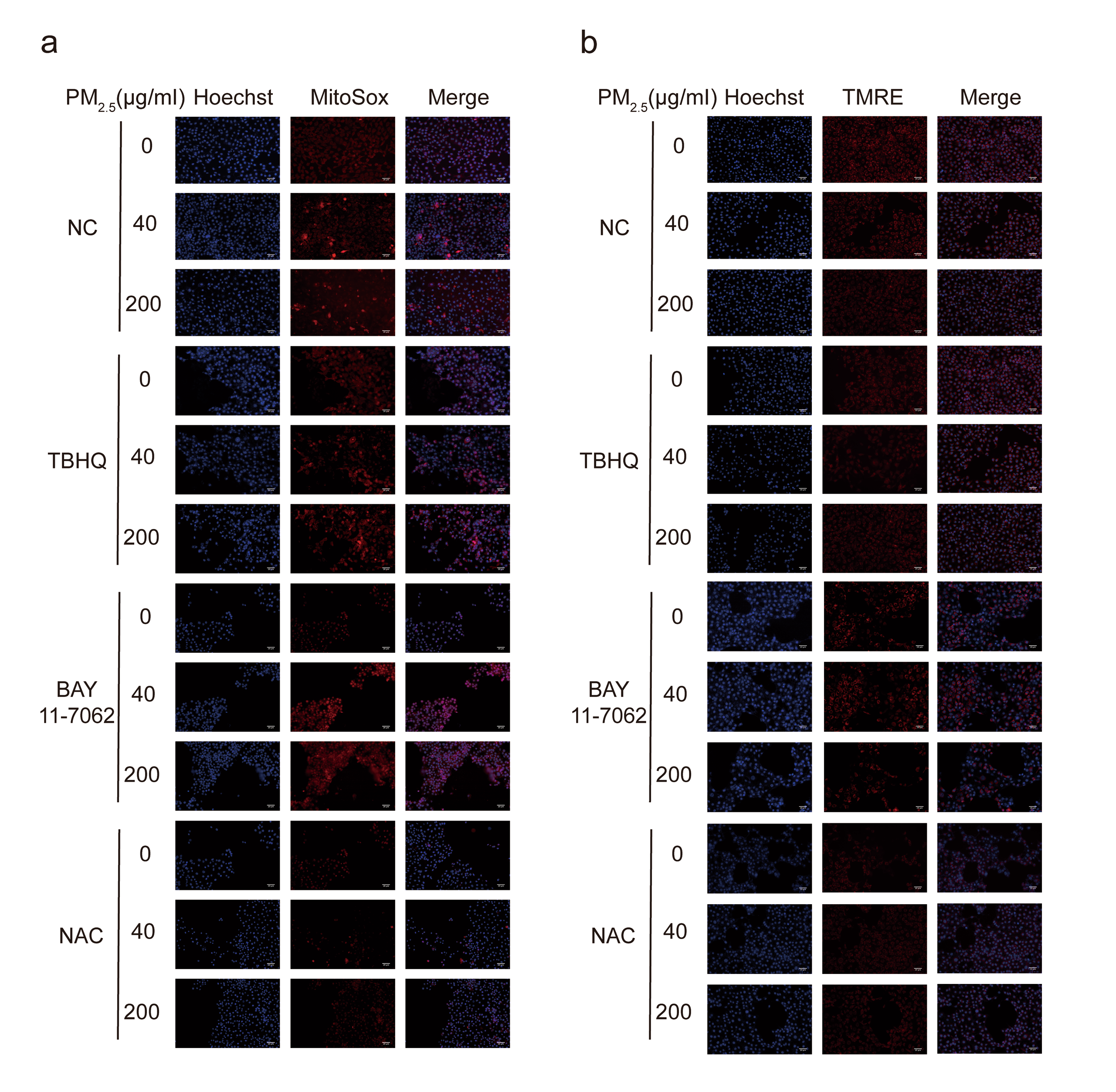

Supplement: Supplementary file 11 — (a) The results of cell mtROS production measured by an inverted fluorescence microscope and a flow cytometer respectively which were treated with MitoSox (5μM) for 40 minutes after exposure (200μg/ml). Cell nucleuses were treated with Hoechst (1μM) for 10 minutes; (b) The results of mitochondrial membrane potential measured by an inverted fluorescence microscope which were treated with TMRE (200nM) for 10 minutes after exposure (200μg/ml). Cell nucleuses were treated with Hoechst (1μM) for 10 minutes. (PNG 3310 kb) [file 10565_2023_9791_Fig11_ESM.png]

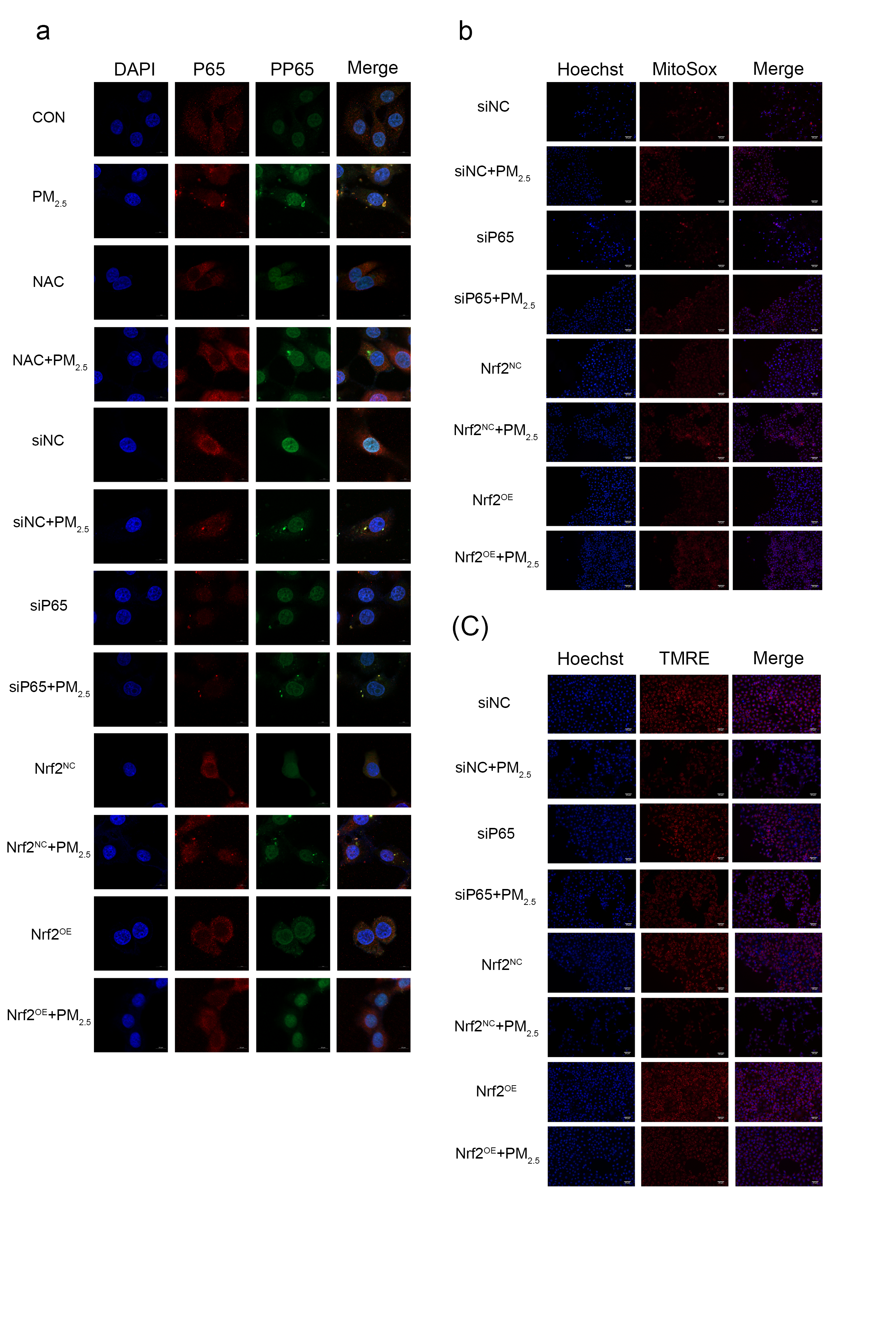

Supplement: Supplementary file 13 — (a) Con-focal pictures of p65 and pp65 proteins immunofluorescence staining after 24hr exposure (200μg/ml); (b) The results of cell mtROS production measured by an inverted fluorescence microscope and a flow cytometer respectively which were treated with MitoSox (5μM) for 40 minutes after exposure (200μg/ml). Cell nucleuses were treated with Hoechst (1μM) for 10 minutes; (c) The results of mitochondrial membrane potential measured by an inverted fluorescence microscope which were treated with TMRE (200nM) for 10 minutes after exposure (200μg/ml). Cell nucleuses were treated with Hoechst (1μM) for 10 minutes. (PNG 3836 kb) [file 10565_2023_9791_Fig12_ESM.png]

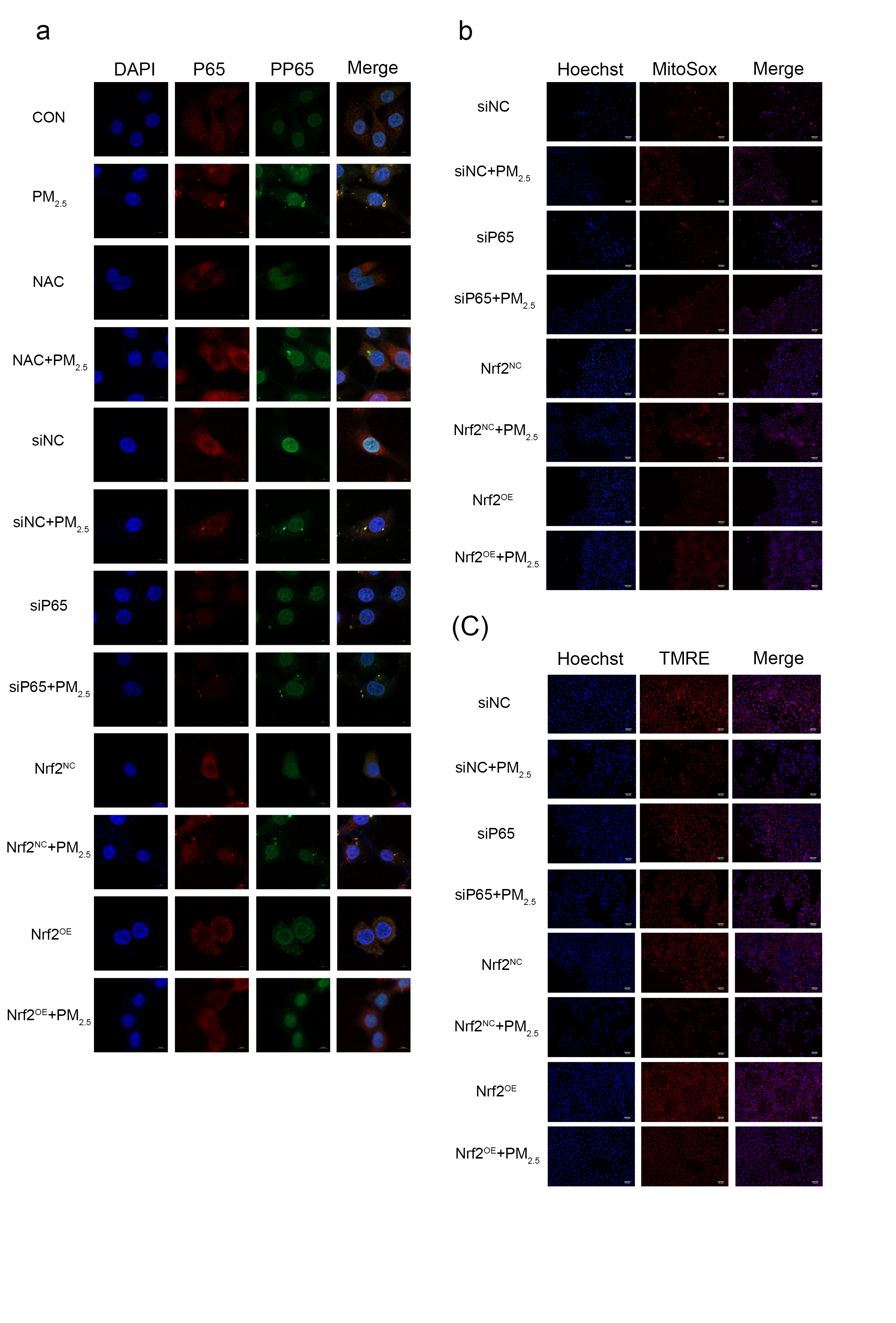

Supplement: Supplementary file 14 — High Resolution Image (TIF 34454 kb) [file 10565_2023_9791_MOESM7_ESM.tif]
